# Risk, Course, and Effect of SARS-CoV-2 Infection in Children and Adults with Chronic Inflammatory Bowel Diseases

**DOI:** 10.3390/children8090753

**Published:** 2021-08-30

**Authors:** Angelica Corrias, Gian Mario Cortes, Flaminia Bardanzellu, Alice Melis, Vassilios Fanos, Maria Antonietta Marcialis

**Affiliations:** Neonatal Intensive Care Unit, Department of Surgical Sciences, AOU and University of Cagliari, SS 554 km 4500, 09042 Monserrato, Italy; angelicacorrias03@gmail.com (A.C.); giamicort@hotmail.it (G.M.C.); ticealy@hotmail.it (A.M.); vafanos@tin.it (V.F.); ma.marcialis@libero.it (M.A.M.)

**Keywords:** COVID-19, Crohn’s disease, ulcerative colitis, ACE2, corticosteroids, biologic drugs, anti-TNF, Infliximab, Tocilizumab

## Abstract

Susceptibility and disease course of COVID-19 among patients with inflammatory bowel diseases (IBD) are unclear and epidemiological data on the topic are still limited. There is some concern that patients with immuno-mediated diseases such as IBD, which are frequently treated with immunosuppressive therapies, may have an increased risk of SARS-CoV-2 infection with its related serious adverse outcomes, including intensive care unit (ICU) admission and death. Corticosteroids, immunomodulators, and biologic drugs, which are commonly prescribed to these patients, have been associated with higher rates of severe viral and bacterial infections including influenza and pneumonia. It is not known whether these drugs can be so harmful as to justify their interruption during COVID-19 infection or if, on the contrary, patients with IBD can benefit from them. As shown by recent reports, it cannot be excluded that drugs that suppress the immune system can block the characteristic cytokine storm of severe forms of COVID-19 and consequently reduce mortality. Another cause for concern is the up-regulation of angiotensin converting enzyme-2 (ACE2) receptors that has been noticed in these patients, which could facilitate the entry and replication of SARS-CoV-2. The aim of this narrative review is to clarify the susceptibility of SARS-CoV-2 infection in patients with IBD, the clinical characteristics of patients who contract the infection, and the relationship between the severity of COVID-19 and immunosuppressive treatment.

**Highlights:**

The questions: Do IBD patients have an increased risk of SARS-CoV-2 infection and/or a worst outcome than general population? Is IBD specific therapy a risk factor for a more severe form of COVID-19?

What we found: IBD patients do not appear to have an increased risk of infection, nor a worse course of COVID-19. The use of corticosteroids is associated with a major risk and more severe forms of infection. Therefore, it would be better to taper the dosage, especially of prednisolone or equivalent, if higher than 20 mg/day. It would also be advisable not to start de novo corticosteroid therapy or eventually to use alternative corticosteroids. Regarding other drugs, the risk of IBD exacerbation seems to outweigh the potential risk associated with COVID-19. This is the case of thiopurines: their use has been associated with more severe viral infections in the past but no data have confirmed that this could also happen in case of SARS-CoV-2 infection. To date, their interruption during the pandemic is not suggested. The case of mesalamines may be similar: although the literature shows plenty of contrasting results, it is suggested to continue the treatment during the COVID-19 pandemic. Further precautions should be considered on a case-by-case basis. Regarding biologics, in our article we underline the presence of various publications bringing out their potential protective role in case of infection. 

What should be further investigated: how some therapies interact with COVID-19 is still not clear. For example, Tocilizumab and anti-TNF may play a role in the dampening of the hyperinflammatory state which characterizes most severe forms of COVID-19. Further studies are necessary to thoroughly understand their role. 

## 1. Introduction

In December 2019 the first case of infection by a new type of coronavirus was documented in Wuhan, China [1]. After a few months, on 11 March 2020, the World Health Organization (WHO) declared a state of pandemic. The disease responsible for the infection is a new coronavirus, which was initially named 2019-nCoV, then changed to SARS-CoV-2 [2]. It is now well known that the presence of comorbidities increases the risk of infection and worsens the outcome in SARS-CoV-2 infection [3,4]. This explains the growing interest regarding the relationship between IBD (inflammatory bowel diseases) and COVID-19. There are several reasons why we might expect a greater risk of SARS-CoV-2 infection and/or a worse course of the disease in patients with IBD: first of all, the weakening of the immune system and the greater risk of infection of these patients, due to the immunosuppressive drugs they take [5]. Secondly, the hyperproduction of cytokines seems to increase the expression of angiotensin-converting enzyme 2 (ACE2) [6]. In these patients, there is also an increased expression of ACE2 in the gut mucosa [7] and an increase of ACE2 serum levels (as well as Ang1–7 and the ACE2: ACE ratio) [8]. This could play a protective role in the blood [9] by acting as a competitive receptor for the virus and thus leading to the reduction of the viral load that would infect the host. 

Since it appears that ACE2 expression is increased in IBD, both mucosal [7] and serum [8], and since IBD patients often take immunosuppressive therapy resulting in deregulation of the immune system, one might expect to see different forms of COVID-19 in these patients compared to the general population. 

The aims of this narrative review are to verify the susceptibility to SARS-CoV-2 infection in IBD patients, the clinical characteristics of infected patients, and the relation between COVID-19 severity and immunosuppressive therapy, through an extensive review of the available literature. 

### Methods

Our bibliographic research is updated to 28 May 2021; we have searched the available literature on Medline, ResearchGate, and Google Scholar, using “COVID-19 and IBD” as key words.

Even though the chosen topic is recent, from last year there have been a constantly increasing interest around the connection between COVID-19 and IBD. There is a large amount of literature available on this subject, but it is not sufficient to guarantee certain answers. We decided to consider clinical trials or articles exposing extrapolated data from registers activated in response to the pandemic. Thus, we selected 38 articles, discussed in the section of our narrative review focused on the available data regarding COVID-19 and IBD. We did not include reviews and other publications concerning, for example, pathogenic mechanisms (e.g., ACE2) or vaccines. These articles were, however, taken into account and discussed in the rest of the review.

**Figure 1:**
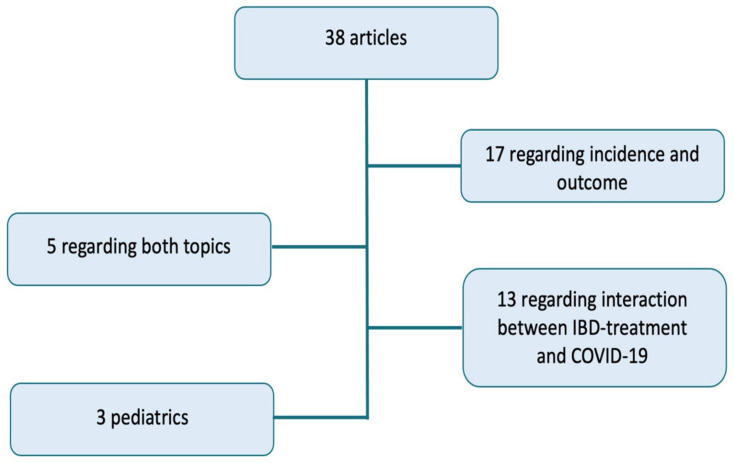
A summary of the 38 analyzed articles: 17 relating to the incidence and outcome of COVID-19 disease in IBD patients, 13 regarding the interaction between IBD-treatment and COVID-19, 5 regarding both these topics, and 3 exclusively children-focused articles.

Figure 1 shows a schematic summary of the 38 analysed articles. A total of 17 articles relate to the incidence and outcome of the infection, 13 relate to the interaction between COVID-19 and IBD treatment. In five articles, both aspects are analyzed. Finally, there are three exclusively pediatric-focused articles. In our discussion, we consider the studies highlighted in Table 1, Table 2, Table 3 and Table 4. Table 1 lists studies that support an increased risk or a worse outcome in IBD patients; on the contrary, Table 2 shows studies according to which incidence and outcomes are better in IBD patients or, at least, similar to general population. Table 3 exposes the three pediatrics studies. Finally, Table 4 shows results of the studies regarding the relation between COVID-19 and IBD drugs.

## 2. Protagonists and Co-Protagonists of The Infection: The Role of ACE2 

The SARS-CoV-2 virus is able to enter human cells thanks to the recognition of the ACE2 receptor [48]. In particular, the virus envelope contains three glycoproteins, including the Spike protein, which represents RBD (receptor binding domain) and the main antigenic site of the virus. This protein is composed of two subunits: S1, receptor recognition site, and S2, which favors the fusion between the viral envelope and the host cell. The cleavage of protein S is regulated by TMPRSS2, a protein consisting of three domains of which the extracellular one has a catalytic function [49]. The virus is able to enter the host cell thanks to the high affinity between RBD and ACE2 (greater than that of the SARS-CoV virus) and thanks to the activation of the furin protease of the virus prior to entry into the host cell, resulting in reduction of dependence on target cell proteases (e.g., TMPRSS2) [50]. Therefore, the simultaneous expression of ACE2 and TMPRSS2 by the target cells is required for viral entry. ACE2 and TMPRSS2 are expressed in different cell types, as alveolar pneumocytes type 2 [51] and in the gastrointestinal tract, where, according to some studies, they would have a greater expression than in the lung [52]. ACE2 is particularly expressed by epithelial cells of the gut, thus leading to the virus entering in these cells and to the consequent infection and damage [53].

**Figure 2:**
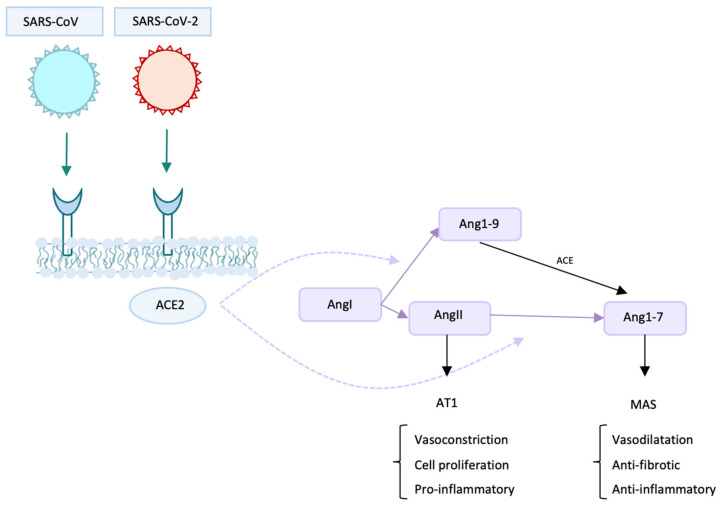
The main roles of ACE2 are shown in the figure. First, it is the cell-receptor for SARS-CoV and SARS-CoV-2. It is also a peptidase: it can create Ang1–9 starting from AngI and Ang1–7 from AngII. ACE2: angiotensin-converting enzyme 2. Ang: angiotensin. AT1: angiotensin II receptor type 1.

ACE2 is also expressed in the testes, kidney, heart, and thyroid [54]. This would explain, for example, the presence of gastrointestinal disorders and renal dysfunction in patients with COVID-19 [55,56]. ACE2 has different functions: besides being the receptor for SARS-CoV and SARS-CoV-2, it is a peptidase and by its activity it can form, starting from Ang I, both Ang 1–9 (which can then be converted into Ang 1–7 by ACE enzyme or other peptidases) and Ang 1–7 [57]. Ang 1–7 has a vasodilatory, anti-inflammatory, and anti-fibrotic function (Figure 2) [58]. ACE2 is also involved in amino acid uptake in the gut and in the regulation of the intestinal microbiome homeostasis [59]. Another interesting topic is the reported finding of SARS-CoV-2 RNA in stool samples of both children and adults, even after they tested negative using the nasopharyngeal swabs [60]. The finding of RNA does not give certain information about the possibility of transmission, since its origin is unpredictable (saliva, exudate). However, there is growing evidence that SARS-CoV-2 may be capable of spreading through fecal-oral transmission. For example, a published study by Xiao et al. reports the presence of infectious virus in feces [61]. From this point of view, the major infection risk may be represented by people with mild nonspecific intestinal symptoms, who could easily transmit the virus [62]. It represents a very complex topic and no certain answers have been found; therefore, more studies are needed.

## 3. Is SARS-CoV-2 Infection Incidence Increased in Inflammatory Bowel Diseases Affected Patients? 

As mentioned above, Table 1 shows studies that conjecture a major incidence and a worse outcome in IBD patients; Table 2 lists studies that do not support this hypothesis. According to the articles we analyzed, susceptibility to SARS-CoV-2 infection does not seem to be increased in patients with IBD compared to the general population (see Table 1 and Table 2). In some cases, on the contrary, these patients seem to have a lower incidence of infection (see Table 2).

In a wide range of cases, including 20,000 IBD patients, no diagnosis of COVID-19 was made until 8 March 2020 [30]. Another significant result is the absence of COVID-19 diagnoses among 5508 IBD patients monitored in Hong Kong and Taiwan [18]. Again, the documented experience at the “Papa Giovanni XXIII” Hospital (Bergamo, Italy) from 19 February 2020 to 23 March 2020 shows no case of infection among IBD patients. During the same period, 479 patients entered the hospital because of COVID-19 related symptoms [21]. Furthermore, the infection incidence was lower in IBD patients than in general population in other studies [17,19]. There are also several studies that did not find significant differences in the incidence of infection [7,11,20,23,26,28]. 

Studies that reported a higher incidence are essentially three, as showed in Table 1 [10,12,13]. The study of Carparelli et al. involved 600 IBD patients, both children and adults, 25 of whom were diagnosed with COVID-19 (no cases among pediatric patients). The study of Ludvigsson et al. was based on the admission to hospital of patients on whom a molecular swab was subsequently performed for a certain diagnosis. In Marafini et al.’s study [15], the incidence turned out to be numerically but not statistically higher in IBD patients than in general population. Finally, Lodyga et al. [16] speculate a more elevated incidence of infection only on the basis of a greater quantity of antibodies (while the risk of disease severity would not increase: in fact, none of the patients had symptoms).

We decided to sum dates regarding COVID-19 incidence obtained by articles summarized in Table 1 and Table 2. Some works were not included because the aims of their research were different (for example, the analysis of the infection course in positive patients or the study of the serological response). Another reason for excluding articles was the fact they regarded not just IBD patients but also patients with other autoimmune diseases. In Table 1, a diagnosis of COVID-19 was made in 1047 patients among 105,290 IBD patients (0.99%). Differently stated, we can observe a reduced incidence from Table 2 data: 529 diagnoses of COVID-19 among a sample of patients more than doubled compared to Table 1: 261,406 patients with IBD (0.2%).

## 4. Outcome: Which Risks for Inflammatory Bowel Diseases Affected Patients? 

Although some studies have found a higher risk of hospitalization in IBD patients [10,11,13] (Table 1), works that do not support this hypothesis are more numerous. Infection lethality was found to be 1% against 9% of the general population in a study by Allocca et al. [20]. In another analysis [26], mortality was found to be lower compared to general population. 

Regarding symptoms, IBD patients only developed mild symptoms in a study by Scaldaferri et al. [25], and 5 out of 15 positive patients in Nancy and Milan needed hospitalization but no one required ICU (intensive care unit) and no death has been recorded [26]. In Lukin et al. [24] analysis, IBD patients have shown a numerically minor risk (which was not statistically significant) of ICU admission, intubation, and death compared to controls. 

No significant differences between IBD patients and general population were found in various studies [22,27,29].

It is notable that IBD severe activity has been associated with infection risk, but this aspect should be considered with caution since severe flares represented a reason for hospitalization and to undergo a molecular swab [23]. Moreover, IBD severe activity has been connected to severe forms of infection [36]. Ulcerative colitis (UC) has been associated with a higher risk of SARS-CoV-2 pneumonia (*p* = 0.03) [36].

### Gastrointestinal Symptoms

A remarkable aspect is the high frequency of gastrointestinal symptoms, especially diarrhea, in IBD patients infected by SARS-CoV-2. A total of 9 out of 12 positive patients studied by Taxonera et al. [17], as well as 38.6% of the patients of Derikx et al.’s study [11], manifested diarrhea. In 49% of patients analyzed by Viganò et al. [23], diarrhea was the first recorded symptom and in general the number of patients with diarrhea was higher in patients with the infection compared to not infected IBD patients (OR:29, *p* < 0.0001). Gastrointestinal symptoms have been found to be very common: they were recorded in 31% of the patients in a study by Allocca et al. [20] and in 50% of the patients analyzed by Guerra et al. [14]. Lukin et al. [24] demonstrated that IBD patients used to show gastrointestinal symptoms more frequently compared to controls (diarrhea 45% vs. 19%, abdominal pain 25% vs. 5%). A similar result has been found by Singh et al. [27]: IBD patients have shown higher percentage of nausea and vomiting (10.77% vs. 4.31%, *p* < 0.01), diarrhea (8.19% vs. 5.14%, *p* < 0.01), and abdominal pain (7.75% vs. 2.7%, *p* < 0.01) compared to COVID-19 non IBD patients. 

## 5. Inflammatory Bowel Diseases, COVID-19 and Children

The studies carried out exclusively on the pediatric population deserve a separate mention, since they have shown even more promising results, which are summarized in Table 3. The incidence rate of COVID-19 in children appears to be lower: in a study by Carparelli et al. [10], among 600 analyzed patients, including pediatric patients, COVID-19 was diagnosed in 25, none of whom <18 years. In most cases, symptoms were absent or mild. No death was recorded. In a study by Brenner et al. [32] the treatment with sulfasalazine/mesalazine (57% of hospitalized patients vs. 21% of non-hospitalized) and the use of corticosteroids (29% vs. 8%) were associated with the risk of hospitalization. Additionally, in pediatric patients, similarly to what was found in adults, the use of TNF antagonists (tumor necrosis factor) alone was associated with a lower probability of hospitalization (7% of hospitalized patients vs. 51% of non-hospitalized).

A publication by Turner et al. [33] underlines the importance of maintaining the underlying IBD treatment. In fact, the risk of inappropriate management of IBD therapy is substantial, as demonstrated by the increased IBD exacerbations in China and South Korea. In fact, data recorded from January 20 to March 20, 2020, show that 233 patients should have had Infliximab infusion during this period, but 28% was postponed and 0.9% cancelled. Of the 66 patients who did not assume the therapy correctly, 14 had an exacerbation, of which 10 required hospitalizations. Conversely, among 1431 pediatric patients with IBD over the same period, only 17 (1.2%) had a flare-up of the disease. The same article reports the South Korea experience: until March 20, 2020, a diagnosis of COVID-19 was made in 525 patients with an age of 19 or less. The indication was to continue therapy without any modifications. However, 13 patients postponed anti-TNF therapy due to fears related to the virus; of these, 3 (23%) had a worsening of the underlying disease.

**Figure 3:**
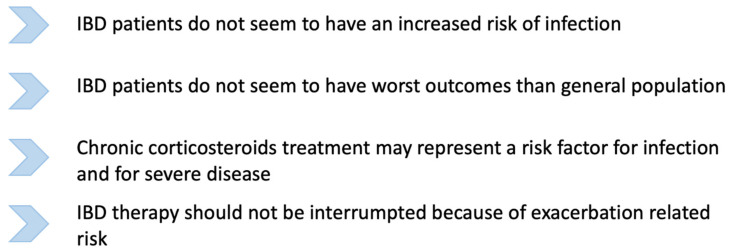
The main findings of our review are summarized in the figure.

A study by Sansotta et al. [34] highlights that, although all patients were instructed to continue therapy, none had a severe course and there were no cases of hospitalization. During this period, two patients experienced fever and gastrointestinal symptoms. After accessing the hospital and after the molecular swab, the results of which were negative, an IBD exacerbation in one case and a Salmonella infection in the other were diagnosed. It is remarkable to note that, although COVID-19 can present itself with gastrointestinal symptoms, all other possible causes must be taken into account. The results of our review are briefly summarized in Figure 3.

## 6. Inflammatory Bowel Diseases and ACE2 

There are many reasons why we might expect an increased risk of SARS-CoV-2 infection and/or a worse course of the disease in IBD patients, as already mentioned in the introduction. There is conflicting evidence regarding the effective increased expression of ACE2 and TMPRSS2 in patients with IBD. In fact, it has been found that ACE2 levels would be down-regulated during inflammation at the level of ileum of patients with CD (Crohn’s disease), but not in the colon of patients with UC [63].

Another study showed that the expression of ACE2 would be increased in the inflamed colon and rectum of IBD patients compared to the non-inflamed one of patients with disease in remission and in controls, while at the ileal level the expression would be reduced [64].

Regarding the increase in ACE2 seric level and its potential protective role, it has been demonstrated, in vitro, that human recombinant ACE2 inhibits the attack of the virus on the cell, depending on the amount of virus and the dose of recombinant human ACE2, thus establishing a dose-dependent mechanism [65]. The first patient that has been treated with hrsACE2 is a 45-years-old woman. She manifested cough, asthenia, myalgia, fever, dyspnoea, nausea, diarrhea, areas of bilateral consolidation on lung RX, and the molecular swab for SARS-CoV-2 was found to be positive. Treatment with hydroxychloroquine and nadroparin was then started. Following the radiographic progression and the worsening of patient’s symptoms, which required intubation, treatment with hrsACE2 was then started, with intravenous infusions at a dosage of 0.4 mg/kg for 5 min twice a day. After the first administration, body temperature returned to normal in a few hours and there was a sharp reduction in angiotensin 2. HrsACE2 administration was continued for 7 days. The course was complicated by methicillin-sensible *S. aureus* bacterial pneumonia and *Enterobacteraerogenes* bacteraemia with the subsequent need for antibiotic therapy (firstly cefuroxime, then linezolid and aztreonam). The patient was then extubated on day 21 and discharged on day 57 from the onset of symptoms (after a rehabilitation period for the myopathy).

Therapy with hrsACE2 resulted in a reduction in IL-6, IL-8, and ferritin. TNF-α and CRP (C-reactive protein) underwent an initial increase (probably due to bacterial infection), followed by a clear reduction. What is most surprising is the reduction in SARS-CoV-2 copies detectable in plasma: 2500 copies/mL the day the therapy was started, 270 copies/mL the next day; subsequently they became undetectable [66]. The administration of hrsACE2 is particularly effective in the transformation of angiotensin 2 into Ang1–7. This may have a key role in therapy since angiotensin 2 infusion has been associated with an increased thrombotic risk and with increase in IL-6 levels [67,68]. On the contrary, and as already mentioned, the increase in Ang1–7 (which represents an alternative pathway to Ang2) determines anti-inflammatory and anti-fibrotic effects. 

## 7. Drugs and Inflammatory Bowel Diseases: What Have We Learned?

The relationship between drugs used to treat IBD and COVID-19 is complex and not yet fully understood. The main results of the studies involved in our review are summarized in Table 4.

Most of the articles we analyzed agree that the greatest risk both in increasing the incidence of infection and in worsening symptoms is related to the use of corticosteroids. In fact, steroid therapy would increase the risk of needing oxygen therapy [47] (*p* = 0.007), the risk of developing a severe form of disease [27,32,46], and even the risk of hospitalization (*p* = 0.015) [20]. 

In a study by Agrawal et al. [43] the use of Vedolizumab has been associated with a higher risk of hospitalization (but not of severe COVID-19 forms) compared to anti-TNF monotherapy, although it is considered safe. The explanation for this phenomenon probably lies in the fact that patients treated with Vedolizumab have shown a greater tendency to develop gastrointestinal symptoms during infection, especially those with IBD in a remission phase [43]. 

The finding of more severe forms of SARS-CoV-2 disease in IBD patients undergoing thiopurine therapy compared to patients with anti-TNF therapy may be due to a negative effect of the former or a protective role of the latter. The possibility of interrupting thiopurine therapy in patients at high risk for COVID-19 with disease in remission phase on therapy with thiopurine + anti-TNF [35] has been proposed.

Data concerning the use of 5-ASAs are conflicting. Ungaro et al. [35] have associated the use of mesalamine/sulfasalazine with severe forms of infection compared to patients with different therapies. However, this supposed association remains just a hypothesis which needs to be confirmed and at the moment there is no indication to interrupt the treatment. A connection between the use of mesalazine and severe infection disease has also been noticed by Brenner et al. [32]. In contrast, Khan et al. [46] found no differences between mesalamine and anti-TNF treatment in a sample of 649 patients with IBD and COVID-19.

However, all studies seem to agree on the importance of not interrupting therapy (except, maybe, for corticosteroids) because of the exacerbations related risk. 

Biological drugs, which are considered potentially protective, and especially anti-TNFs, are placed outside the box. Allocca et al. [20] found a reduction in the risk of pneumonia and hospitalization (OR 0.15 and 0.31) in patients receiving monoclonal antibodies therapy. Patients on anti-TNF therapy have developed severe forms of the infection to a lesser extent than the others in a study by Ungaro et al. [35]. Moreover, biologics have shown a protective role against the infection (1.6% vs. 7.6%) in a study by Bezzio et al. [36]. 

### 7.1. Corticosteroids: Instruction for Use

The use of corticosteroids is a very debated topic. Although these, and in particular dexamethasone, are used in the treatment of severe forms of infection associated with respiratory distress [69], their use must be reserved for the second phase of the disease, when the damage mediated by the hyper-inflammatory state prevails. On the contrary, they could increase replication and viral load if used immediately [70,71]. It has been demonstrated that the chronic use of systemic corticosteroids leads to immunosuppression. Their anti-inflammatory activity affects both the innate and the adaptive immune response. Corticosteroids specifically decrease the activity of neutrophils, macrophages, monocytes, and plasma cells. This explains how people under this therapy could be at higher risk of infection and particularly of severe forms of infection [72].

Previous studies on MERS, SARS and influenza viruses have shown unfavorable outcomes in patients receiving corticosteroids [73,74].

We have analyzed several studies that associate the use of corticosteroids with a greater risk of infection or worse outcome of the disease.

Since corticosteroids seem to increase not only infection risk, but particularly the risk of undergoing severe forms of infection, which may lead to need for oxygen therapy, hospitalization, and even ICU admission, we looked for publications giving advice to follow during the pandemic. The precautions that should be taken for steroid therapy could be minimize their use, avoid starting new therapies, and taper down the dose if it is higher than 20 mg/day of prednisolone or equivalent, always taking into account the disease activity. In case of acute, severe form of UC, corticosteroid treatment could be started after urgent molecular swab or after excluding COVID-like symptoms [9].

The British Society of Gastroenterology suggests alternatives to prednisolone, especially if a high-dose is assumed: budesonide (9 mg per day for 8 weeks) or beclometasone (5 mg per day for 4 weeks) for patients with a flare of UC. Other suggestions are exclusive enteral nutrition (EEN) for CD exacerbations or budesonide (9 mg per day for 8 weeks) for active CD in the small bowel [75].

Some alternatives to corticosteroids use, in particular regarding CD in children, are thiopurines (to maintain the remission) or EEN (which has been demonstrated to have the same efficacy of corticosteroids in remission induction) [76].

### 7.2. Thiopurines: Should More Attention Be Paid in Elderly Patients?

Thiopurines are associated with more of a major risk of viral infections than anti-TNFs [77,78]. Severe forms of infection caused by Epstein-Barr virus, cytomegalovirus, and varicella zoster virus have been related to the use of these drugs. The risk related to the intake of thiopurines should be taken into consideration, not so much in young patients without other diseases, but rather in elderly individuals with comorbidities. In fact, it must be considered that thiopurines are associated with the risk of lymphoma and they also have limited efficacy, when used alone, in CD treatment [79]. Moreover, the immunosuppressive effect, even if therapy is interrupted, could persist for weeks or months [75]. Given the fact that more severe viral infections have been documented in the past in patients on thiopurine therapy, this possibility should be considered for SARS-CoV-2 infection. However, this remains a hypothesis that no data seem to confirm at the moment.

Ungaro et al. [35] propose the possibility of interrupting thiopurine therapy, if associated with anti-TNF, in patients at high risk for COVID-19. Nevertheless, currently, discontinuation of therapy is not indicated except in particular conditions that must be assessed on a case-by-case basis. 

### 7.3. Mesalamines: A Summary of Available Literature So Far 

Data concerning 5-ASA are definitely not conclusive but provide a basis to hypothesize that their interruption during the pandemic would not be the best decision. As mentioned above, some analyzed articles suppose that the use of mesalamine is connected to more severe forms of COVID-19 disease [35,44]. Other studies do not find any differences between patients treated with mesalamine and with other therapies (anti-TNF). In conclusion, although results of our review are conflicting, an increased risk of infection related to their use has not been demonstrated at present. Therefore, current indications remain to continue 5-ASA therapy, even in case of infection [80].

### 7.4. Biologic Drugs Protective Role

**Figure 4:**
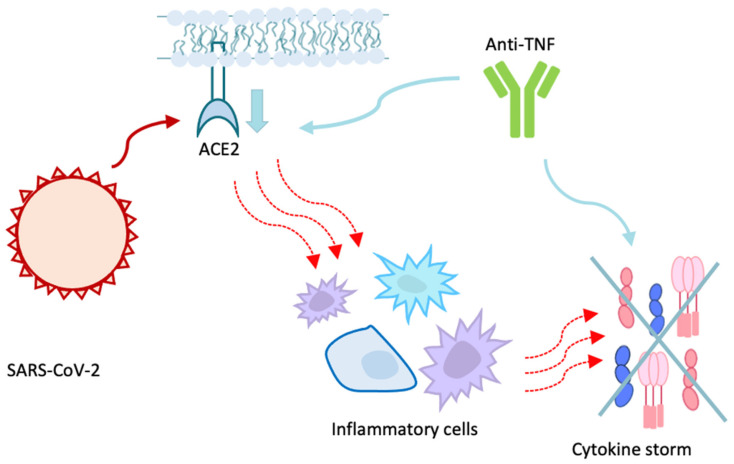
The potential protective role of anti-TNFs against COVID-19 is synthetically explained in this figure. Anti-TNFs have been shown to downregulate ACE2, leading to a most difficult virus entry into the cells. These drugs also have an anti-IL-6 activity, and this could contribute to the dampening of cytokine storm which is activated in most severe forms of COVID-19. ACE2: angiotensin converting enzyme 2. TNF: tumor necrosis factor.

One aspect that needs to be underlined is biologics, and in particular anti-TNF’s activity, since they seem to have a protective role. Regarding the mechanism by which anti-TNFs would play this protective role (Figure 4), one proposal is that they would reduce IL-6 levels in patients with CD [81]. IL-6 has a pivotal role in SARS-CoV-2 infection: following the ACE2—Spike protein binding, the host’s immune response is activated, with the accumulation of inflammatory cells and consequent activation of the cytokine storm. This storm prolongs the hyperinflammatory state, leading to severe outcomes such as pneumonia, acute respiratory distress and multi-organ failure (MOF) [6].

The use of IL-6 concentration as an indicator of the extent of the inflammatory response to SARS-CoV-2 infection has also been proposed [82]. The effect of anti-TNFs on IL-6 could therefore explain their potential protective role [83].

Another proposed mechanism is the ACE2 down-regulation [84]. In a study by Li et al. [85] it is proposed that this effect could manifest itself in responders but not in non-responders to the anti-TNF therapy. In a cross-sectional study, no differences were found in ACE2 and TMPRSS2 expression in the ileum between patients receiving anti-TNFs and controls. Conversely, ACE2 and TMPRSS2 levels in the colon were higher in patients under anti-TNFs, particularly in the inflamed rectum [64]. This could be related to a major infection risk. However, this has not been demonstrated while, on the contrary, the protective role performed by the anti-inflammatory action of these drugs seems to be predominant. Since these data are based on a cross-sectional study, which has some limits, previously published data were later analyzed to evaluate eventual alterations in ACE2 and TMPRSS2 expression at the ground zero and after 6 weeks of treatment. It was found that, after 6 weeks of Infliximab, ACE2 expression was decreased, particularly in responders [64]. 

Based on current knowledge, therefore, anti-TNF therapy should not be interrupted during COVID-19 infection [81].

In a study by Kennedy et al. [39], the use of Infliximab seems to be associated with a low antibody response. This may be due to the anti-TNFs activity. In fact, TNF has several pro-inflammatory functions, and it can stimulate lymphocytes B to product immunoglobulins [86]. 

Moreover, in a study conducted by Huang et al. [87], among 41 hospitalized patients with COVID-19, ICU admitted patients used to have higher plasmatic levels of IL2, IL7, IL10, GCSF, IP10, MCP1, MIP1A, and TNFα compared to other patients [87]. This suggests a potential protective role of anti-TNFs. Fieldmann et al. [88] proposed the use of anti-TNFs in case of moderate/severe forms of infection. The treatment could be started in patients that require hospitalization and oxygen-therapy as soon as possible. Additionally, their use outside the hospital for elderly patients with comorbidities, who run a high risk of severe disease, is proposed. 

We want to highlight a case report of a 14-years-old patient with ileal and peri-anal CD. This child presented with fever, abdominal pain, tachycardia, and maculopapular erythematous rash initially on the face, then widespread. He also presented increased inflammatory markers (such as IL6, IL8, and TNFα) and hepatic enzymes. After an initial treatment based on antibiotic for a peri-anal abscess, hydroxychloroquine, azithromycin, and enoxaparin for COVID-19, symptoms did not seem to improve. The child still manifested fever, tachycardia, and fluid refractory hypotension. The therapy was then modified, but without any improvement. It was therefore decided to start Infliximab therapy with 10 mg/kg/day on day 8, due to its possible dual action and rapid clinical deterioration. Within a few hours, a normalization of inflammatory markers and improvement of clinic were noticed. A second Infliximab infusion was made after 5 days (10 mg/kg), just before the patient’s discharge. After two weeks, it was found a complete both clinic and laboratoristic resolution. It is important to underline that TNF α and IL6, which are increased in IBD patients, have been also associated with severe forms of COVID-19. The treatment with Infliximab, already used in children with CD, was revealed to be effective on both fronts. The use of anti-TNFs especially for MIS-C (multisystem inflammatory syndrome in children) requires further investigation [89]. 

Vedolizumab has a specific intestinal action. It does not alter pulmonary or systemic response to SARS-CoV-2 [33]. Similarly, Ustekinumab (anti IL12/12) has very low systemic activity and this would make it safer in case of COVID-19 [90]. In particular, elderly patients with comorbidities and a high risk of infection could take Vedolizumab or Ustekinumab if they need biologic therapy [91]. 

In contrast to what was found regarding Infliximab, after 6 weeks of Vedolizumab no differences were found in the expression of ACE 2 and TMPRSS2 [64]. The same study also analyzed Ustekinumab effect. With its use, ACE2 expression increased in the inflamed gut, while in placebo-treated patients the expression of ACE2 was reduced. Therefore, the effects of these drugs are complex, region-specific, and drug-specific [64]. 

### 7.5. New Drugs

Janus kinase (JAK) are enzymes with a central role in the JAK/STAT signaling pathway. As a result of the link between type I or II cytokine receptor and its ligand (interleukins, interferon, colony stimulating factor (CSF), and some hormones), JAKs phosphorylate the intracellular domain of the receptor with consequent call and activation of STAT transcription factors, which will regulate gene transcription [92]. 

JAK’s inhibition represents a new approach for IBD treatment. Their inhibition damages the virus entering and, consequently, the cytokine storm. Currently, some clinical trials are using JAK’s inhibitors to treat COVID-19 [93,94]. Baracitinib has a double action. In addition to the inhibition of numb-associated kinase (NAK), it has an anti-inflammatory activity, with a reduction of the cytokine storm and its consequent possible utility in COVID-19 severe forms. Tofacitinib has been associated with an increased risk of complications from viral infection, particularly HZV [95]. Although data are currently limited, it is recommended to continue the treatment in IBD patients, amongst whom it is widely used for the treatment of UC [96]. 

Tocilizumab, an IL-6R blocker, is not currently recommended but it is under study for IBD treatment. It could be useful in the treatment of severe forms of COVID-19, where, as already mentioned, the systemic damage is mainly mediated by the pro-inflammatory state, in which IL-6 plays a fundamental role [82]. 

A clinical trial was conducted in China on 21 patients who met the disease severity criteria and were treated with Tocilizumab (in addition to previous traditional treatment with Lopinavir/Ritonavir, IFN-α and ribavirin, glucocorticoids, and oxygen therapy if needed). After the treatment, body temperature improved, as did the respiratory function. A reduction of oxygen flow necessary to maintain a stable saturation was noticed. Lymphocyte values returned to normal in 52.6% of patients in 5 days of therapy, as did CRP values. CT pulmonary opacities resolved in 90.5% of patients. IL-6 levels remained high for some days because of the receptor block, but they subsequently returned to normal values. No adverse effect or pulmonary superinfection have been recorded. However, this study only considers a few patients. Further validation and explanations are needed [97]. 

In a study conducted on 389 hospitalized patients with COVID-19, amongst whom 249 were treated with Tocilizumab and 128 with placebo, the group treated with Tocilizumab showed lower rates of mechanical ventilation and death within 28 days (12% vs. 19.3%, *p* = 0.04) [98]. 

A multicenter study including 3924 critical patients found a reduction in the risk of in-hospital mortality in those who received Tocilizumab in the first two days of ICU admission compared to other patients. In fact, the death rate was 28.9% among Tocilizumab-treated group and 40.6% among those who did not assume the drug [99]. 

Even in this case, however, the results of the studies are conflicting. A double-blind randomized trial involved 243 patients with severe COVID-19. One group was given Tocilizumab in addition to normal therapy; placebo was added to the second group. Tocilizumab has not demonstrated effects in preventing intubation or in reducing mortality (HR: 0.83, 95% CI 0.38–1.1, *p* = 0.64) [100]. 

## 8. What to Do in Case of SARS-CoV-2 Infection: What British Society of Gastroenterology Suggests 

There is not certain information regarding the continuation or discontinuation of therapies so far. It definitely represents a very debated topic with contrasting opinions that still requires additional studies. We decided to report, besides the new scientific studies results, the British Society guidelines (which particularly regard what to do in case of SARS-CoV-2 infection and not during the pandemic in general) to have a more practical guide.

In case of COVID-like symptoms, the British Society of Gastroenterology recommends, in addition to general isolation measures, to discontinue the following therapies: anti-TNFs, Ustekinumab, Vedolizumab, Thiopurine, MTX, calcineurin inhibitors, JAK inhibitors, mycophenolate mofetil, and thalidomide. Concerning corticosteroids treatment, the dosage should be quickly tapered (10 mg/week) but it should not be abruptly stopped. It is important to take into account the risks associated with a too rapid reduction in dosage. The treatment could be resumed, after consultation with the referring physician, two weeks after the resolution of symptoms [75]. A different situation is that of 5-ASA, which use can be continued even during the infection [80]. 

A proposed alternative is to discontinue the therapy in case of infection with the presence of symptoms, while patients with diagnosed infection but who remain asymptomatic should hold thiopurines, Methotrexate, and Tofacitinib and postpone treatment with biologics for two weeks of monitoring any symptoms of COVID-19 [101]. 

The recommendations presented by Siegel et al. [102] regarding when to start post-COVID-19 therapy again are based on the IOIBD (International Organization for the Study of Inflammatory Bowel Disease). There are two possible strategies: one based on the symptoms and one on the molecular test. Concerning the first strategy, the therapy could be restarted at least after 3 days after the fever resolution, the improvement in respiratory symptoms, and at least 10 days after the first symptoms appear. On the other hand, there is the molecular-based strategy. In this case, the clinical criteria must be met, but the negativity of two molecular tests performed at least 24 h apart from each other is also required [102]. 

## 9. Vaccines against COVID-19 and Inflammatory Bowel Diseases: A New Challenge

COVID-19 vaccination is a current topic of great interest both in general population and in IBD patients. However, new aspects come into play in patients with IBD, which are still under study and need further clarification. Currently, there are different approved vaccines to contrast COVID-19 pandemic; however, IBD patients, and in general patients undergoing immunosuppressive therapy, have not been included in studies for the vaccines’ approval. Therefore, it remains unclear if their efficacy is comparable to that found in the general population [103]. In fact, studies carried out on the efficacy of other vaccines have found a lower immunological response in patients treated, in particular, with anti-TNFs. This is the case, for example, of what has been found in a pediatric study on the efficacy of the flu vaccine: patients receiving Infliximab and immunomodulators showed a reduced immunological response compared to other therapies (thiopurines, corticosteroids, mesalazine) [104]. Similarly, a reduced immunological response has been found in patients receiving Infliximab compared to patients receiving mesalamine in a study based on the efficacy of the pneumococcal vaccine [105]. Furthermore, according to an aforementioned study, patients receiving Infliximab showed lower seroconversion rates than patients receiving Vedolizumab, despite the similar incidence of COVID-19 symptoms [39]. However, the current indication is to vaccinate IBD patients, without preference for a particular vaccine. In fact, as suggested by the British Society of Gastroenterology, vaccination is safe, and the only risk is represented by the possibility of a sub-optimal immunological response in patients under immunosuppressors. This risk must be further investigated, and new solutions must be found [106,107]. 

Another aspect that has been much debated is the risk of hypersensibility reactions, particularly regarding Pfizer-BioNTech and Moderna vaccines (but also Johnson & Johnson). Polyethylene glycol (PEG) ought to be responsible for these reactions, even though it still remains unclear. PEG’s structure is similar to that of polysorbate, and this leads to possible cross-reactions that could explain the Jannsen’s case of hypersensibility. It is noteworthy that various biologics contain polysorbate 80 as excipient: it is the case of Infliximab, Adalimumab, Golimumab, Natalizumab, Ustekinumab, and Vedolizumab [108]. In fact, several immediate hypersensitivity reactions are reported after taking Infliximab [109]. The only current contraindication to vaccination is a history of a severe and immediate allergic reaction to that vaccine or a reaction to a component of the vaccine. In case of biologics, however, most of the time patients who developed allergic reactions do not test for the allergy and so there is no certainty as to what is actually responsible for the reaction. An interesting possibility could be a skin test to highlight the presence of a possible IgE-mediated reaction [110]. 

In any case, allergy to PEG is currently not an absolute contraindication for the Janssen vaccine, just as allergy to polysorbate 80 is not an absolute contraindication for the administration of mRNA vaccines [111]. 

Finally, it must be considered that psychological aspects related to vaccination, which relates to everyone but especially people with chronic diseases. In a recent French study, 104 IBD patients were asked to answer a questionnaire. In total, 54.8% of them expressed the intention to receive anti-COVID-19 vaccination as soon as possible, and this percentage was comparable to that of the general population in France [112]. 

Although a good percentage of patients has understood the importance of vaccination, these numbers are still not sufficient, and a responsibility of the healthcare workers should be to ensure adequate information for the general population.

## 10. Conclusions

The relationship between COVID-19 and IBD is garnering growing interest, but several questions are yet to be clarified, through future specific studies involving greater cohorts of patients. It does not appear that IBD patients face a greater risk of infection or a more severe course of the disease. However, since the results of the studies published so far are often discordant, further analyses are required to achieve greater certainties.

Currently, recommendations suggest not to interrupt IBD therapy during the pandemic, since the risk of exacerbations outweighs the risk of any COVID-19 complications [12,33].

Such a recommendation is especially strong in children. During the first wave of COVID-19 pandemic, 21–23% of pediatric patients that had interrupted or temporarily suspended the biologic treatment experienced flare-ups of the disease [113]. The same applies for adults, in which therapeutic approaches must be analyzed on a case-by-case basis and further investigation is needed to clarify the potential role of drugs such as anti-TNF and Tocilizumab in SARS-CoV-2 infection.

However, the discussion regarding the relationship between severity of COVID-19 infection and immunosuppressive therapy, as well as the indications on therapy suspension or continuation during COVID-19, should take into account some discordant results emerging between the various studies reviewed. The published literature does not provide univocal results in this sense, so we tried to report all the different aspects that the analyzed articles underline.

The conclusions we made are deduced from the whole literature presented, but they are not based on a statistical analysis of data. In our opinion, the analyzed literature provides very interesting information about SARS-CoV-2 infection in IBD patients and the possible role of different therapies. However, we must consider that these represent preliminary results and furthermore some limitations should be acknowledged; the analyzed studies are not homogeneous and easily comparable regarding methodologies, enrolled patients, diagnostic methods, considered period, and outcomes and, therefore, it is highly difficult to find similar and comparable investigations. COVID-19 has provided several questions and more studies are needed to fully understand the virus’ behaviour in IBD patients. 

## Figures and Tables

**Table 1:** COVID-19: increased SARS-CoV-2 infection risk in IBD patients.

Author	Patients	Diagnostic Method	Geographic Area	Time/Duration of the Study	Results
Carparelli et al., 2021 [10]	IBD: 600 COVID-19: 25	Molecular swab (PCR) or serological test	Foggia (Italy)	Until January 2021	COVID-19 incidence in IBD patients (4.1%) > incidence in general population (2.8%) Hospitalization in IBD patients (12%) > hospitalization in Italian population (4.8%)
Derikx et al., 2021 [11]	IBD: 34,763 COVID-19: 100 (0.29%)	PCR 96/100 TC 3/100 Serological test and symptoms 1/100	Netherlands	From March to June 2020	COVID-19 incidence in IBD patients (287.6/100,000) comparable to general population (333/100,000), *p* = 0.15 Mortality in IBD patients (37.3/100,000) comparable to general population (44.9/100,000), 0 = 0.51 Among 100 infections, 59 hospitalizations and 13 deaths Hospitalization risk in IBD patients (177.2/100,000) > general population (84.5/100,000), *p* < 0.01
Rizzello et al., 2020 [12]	IBD: 1158 COVID-19: 26 (2.2%)	Molecular swab (PCR)	Italy	10 March 2020–10 June 2020	COVID-19 incidence in IBD patients (22.4/1000) > incidence in Italy (3.91/1000, respectively 9.01, 6.27, and 7.10/1000 in Lombardy, Emilia Romagna, and Piedmont)
Ludvigsson et al., 2021 [13]	IBD: 67,292 (of which 6569 < 18aa) COVID-19: 811 (1.21%) Controls: 297,910 (of which 30,891 < 18aa) COVID-19: 2890 (0.97%)	Laboratory diagnosis	Sweden	01 February 2020–31 July 2020	COVID-19 incidence in IBD patients (5.4/1000) > controls (3.4/1000) 1/185 IBD patients that required hospitalization < 1/295 in controls (179 vs. 500): the risk of hospitalization is increased 43% in IBD patients (0.27% vs. 0.17% in controls) No increased risk of severe forms (ICU or death)
Guerra et al., 2020 [14]	IBD: 805 COVID-19: 82	PCR 28 patients Clinic 54 patients (highly suspected)	Madrid (Spain)	Until 27 May 2020	COVID-19 incidence: 10.2% 79.3% mild symptoms, 12.2% moderate symptoms, 8.5% severe symptoms, 1 death
Marafini et al., 2020 [15]	IBD: 672 COVID-19: 3	Molecular swab (PCR)	Tor Vergata, Rome (Italy)	Until 30 April 2020	COVID-19 incidence in IBD patients (4.46/1000) > Italian population (3.41/1000) *p* = 0.5
Lodyga et al., 2021 [16]	IBD: 432 Controls: 432	Serological test	Warsaw, Lodz and Poznan (Poland)	01 May 2020–15 June 2020	IgG: 4.6% of IBD patients and 1.6% of controls, *p* < 0.05 IgA + IgM: 6% of IBD patients and 1.1% of controls, *p* < 0.05

IBD: inflammatory bowel disease. PCR: polymerase chain reaction. TC: computed tomography. ICU: intensive care unit.

**Table 2:** COVID-19: are IBD patients protected from the infection?

Author	Patients	Diagnostic Method	Geographic Area	Time/Duration of the Study	Results
Taxonera et al., 2020 [17]	IBD: 1912 COVID-19: 12	Molecular swab (PCR)	Madrid (Spain)	Until 08 April 2020	COVID-19 incidence in IBD patients (4.9/1000) < general population (6.6/1000), OR:0.74, *p* < 0.001 Mortality in IBD patients (0.82/1000) < general population (0.9/1000) but not statistically significative, *p =* 0.36
Mak et al., 2021 [18]	Hong Kong IBD: 2954 Taiwan IBD: 2554	Molecular swab (PCR)	Hong Kong and Taiwan (China)	21 January 2020–15 April 2020	0 COVID-19 cases among IBD patients General population: 1017 cases in Hong Kong, 429 cases in Taiwan
Maconi et al., 2020 [19]	IBD: 941 COVID-19: 2 Controls: 869 COVID-19: 10	Molecular swab (PCR) (certain cases) Clinic (highly suspected cases)	Lombardy (Italy)	Until 25 April 2020	Certain diagnosis of COVID-19: 2 IBD patients and 10 controls, *p* = 0.018 Highly suspected COVID-19: 3.8% of IBD patients < 6.3% of controls, *p* = 0.006
Allocca et al., 2020 [20]	IBD: 23,879 COVID-19: 97	Molecular swab (PCR): 64 patients Clinic + contact or radiology: 33 patients (highly suspected)	Italy, United Kingdom, France, Spain, Portugal, Malta, Kastoria, Attica, Greece, Russia, Israel	21 February 2020–30 June 2020	COVID-19 incidence in IBD patients (0.406%) comparable to general population (0.402%) Lethality in IBD patients (1%) < general population (9%)
Norsa et al., 2020 [21]	IBD: 522, of which 59 < 18 aa Controls with COVID-19: 479	Molecular swab (PCR)	Hospital “Papa Giovanni XXIII”, Bergamo (Italy)	19 February 2020–23 March 2020	0 cases of COVID-19 in IBD patients 479 COVID-19 patients accessed the hospital during the same period
Quera et al., 2020 [22]	IBD: 1432 COVID-19: 32	Molecular swab (PCR)	Chile	01 March 2020–31 August 2020	Hospitalization in 4 patients. No death. IBD patients do not have an increased risk of severe symptoms
Viganò et al., 2020 [23]	IBD: 704 COVID-19: 53	Laboratory diagnosis (9 patients, 1.2%) or highly suspected clinic based on WHO criteria (+ contact or flu vaccine)	Lombardy	Until April 2020	COVID-19 incidence in IBD patients (1.2%) comparable to general population (0.81%) Association between IBD severity and COVID-19 (OR:12.6, *p* = 0.01)
Lukin et al., 2020 [24]	IBD e COVID-19: 80 COVID-19 non IBD: 160	Molecular swab (PCR) or highly suspected clinic	New York (USA)	01 February 2020–30 April 2020	Risk of ICU admission, intubation and death resulted minor in IBD patients compared to controls (24% vs. 35%) but the result is not statistically significative (*p* = 0.352)
Scaldaferri et al., 2020 [25]	IBD: 1451 COVID-19: 5	Molecular swab (PCR)	Rome (Italy)	04 March 2020–15 April 2020	Only mild symptoms in positive patients
Allocca, Fiorino et al., 2020 [26]	IBD: 6000 patients COVID-19: 15	Molecular swab (PCR)	Nancy (France) and Milan (Italy)	Since the beginning of pandemic (publication date: 30 April 2020)	COVID-19 incidence in IBD patients (0.0025) comparable to general population (0.0017) Mortality and need for hospitalization higher in general population (13% vs. 5%), 5 hospitalizations, 0 ICU admission 0 deaths
Singh et al., 2020 [27]	IBD: 196,403 COVID-19: 232 Controls: 19,776 COVID-19	Laboratory diagnosis or COVID-19 diagnostic code after hospitalization	USA	26 January 2020–26 May 2020	Risk of severe disease (hospitalization and/or death within 30 days) comparable between IBD patients (56/232) and controls (4139/19,776), RR: 0.93, *p* = 0.66
Gubatan et al., 2020 [28]	IBD: 168 (tested) COVID-19: 5	Molecular swab (PCR)	Northern California (USA)	04 March 2020–14 April 2020	Positivity rate comparable between IBD patients (3%) and general population (2.8%)
Kjeldsen et al., 2021 [29]	132 hospitalized patients for COVID-19 having IBD/RA/AS/psoriasis 2811 controls hospitalized for COVID-19	Hospitalized patients with COVID-19 diagnostic code (from national database)	Denmark	01 March 2020–31 October 2020	No significative differences between the group of patients with underlying diseases and controls in terms of hospital persistence (6.8 vs. 5.5 days), need for mechanical ventilation (7.6% vs. 9.4%), need for CPAP (11.4% vs. 8.8%), in-hospital, within 14 and 30 days mortality (17.4%, 20.5% e 21.2% vs. 15.2%, 18.1% e 19.1%, OR 0.71, 0.70 e 0.68)
Mao et al., 2020 [30]	IBD: 20,000 COVID-19: 0 (the three biggest centers in Wuhan have been analyzed)	Laboratory diagnosis	China	December 2019–08 March 2020	0 COVID-19 diagnosis
Attauabi et al., 2020 [31]	IBD: 2486 COVID-19: 76 COVID-19 general population: 8476 out of 231601 swabs	Molecular swab (PCR)	Denmark	28 January 2020–02 June 2020	Prevalence in IBD patients (2.5%) < general population (3.7%), *p* < 0.01 (with more tests performed in percentage in patients with IBD) Hospitalization in 25% of patients, need for oxygen-therapy in 18.4%, 4 deaths Dyspnea as presenting symptom is a risk factor for access ICU (OR: 19.7)

IBD: inflammatory bowel disease. RA: rheumatoid arthritis. AS: ankylosing spondylitis. PCR: polymerase chain reaction. WHO: World Health Organization. ICU: intensive care unit. CPAP: continuous positive airway pressure.

**Table 3:** COVID-19 and IBD in children.

Authors	Patients	Diagnostic Method	Geographic Area	Time/Duration of the Study	Results
Brenner et al., 2021 (pediatric) [32]	IBD and COVID-19: 209	Laboratory diagnosis	23 countries (SECURE-IBD and COVID-19 Pediatric IBD Porto Group)	Until 01 October 2020	7% hospitalizations, of which 1% mechanical ventilation (sulfasalazine/mesalazine therapy, they developed multisystemic inflammation and superinfection). 0 deaths Hospitalization rate < IBD adult patients (33–66%)
Turner et al., 2020 (pediatric) [33]	PIBD: 102 COVID-19: 8 (6 confirmed)	Laboratory diagnosis in 6 patients Highly suspected clinic in 2 patients	Porto Group-affiliated Pediatric IBD centers in Europe	Until 26 March 2020	Only mild symptoms (fever, cough, ageusia, myalgia, anosmia, asthenia)
		Laboratory diagnosis or clinic suspect	China	Until 20 March 2020	Out of 917 pediatric cases of SARS-CoV-2 infection, none had IBD
Sansotta et al., 2021 (pediatric) [34]	PIBD: 290 COVID-19: 24 (8%)	Clinic in 22 patients Molecular swab in 2 patients	Lombardy (Italy)	21 February 2020–04 May 2020 (lockdown period)	Only 8% of children developed COVID-like symptoms, on which the supposed diagnosis was based given the scarce availability of swabs. 42% thiopurine therapy, 30% salicylates, 16% organic. No severe course or need for hospitalization

IBD: inflammatory bowel disease. PIBD: pediatric inflammatory bowel disease. PCR: polymerase chain reaction.

**Table 4:** IBD-treatment and COVID-19: risks and benefits.

Author	Patients	Diagnostic Method	Geographic Area	Time/Duration of the Study	Clinic Results	Main Purposes of the Study	Therapy’s Effects
Ungaro et al., 2020 [35]	IBD and COVID-19: 1439	Laboratory diagnosis	SECURE-IBD (47 countries)	13 March 2020–09 June 2020	112 patients (7.8%) severe form 82 ICU 66 mechanical ventilations 49 (3.4%) deaths	To evaluate the course of COVID-19 in IBD patients under different therapies	Severe form of disease: patients under anti-TNFs (1.1%) < other patients (4.8%), *p* < 0.001 Anti-TNF are not associated with COVID-19 severe forms (aOR:0.69). Patients under thiopurine or thiopurine + anti-TNFs (9.2% and 8.8%) > patients under anti-TNFs monotherapy (2.2%), *p* < 0.001. Risk of COVID-19 severe form is increased in patients under thiopurine treatment (aOR:4.08) or combined therapy (aOR:4.01). Patients under mesalamine (sulfasalazine (13.9%) > other patients (5.2%), *p* < 0.001 Increased risk of COVID-19 severe forms (aOR: 1.47)
Bezzio et al., 2020 [36]	IBD: 243 COVID-19: 11 (1 confirmed and the other made by contact + clinic)	Molecular swab in 1 patient Clinic (min 3 symptoms) + contact in 10 patients	Italy	10 March 2020–03 May 2020	124 patients on biologic therapy (2 COVID-19) 119 patients not on biologic therapy (9 COVID-19)	To assess the incidence of COVID-19 in IBD patients relating to the use or biologics	COVID-19 incidence: patients on biologic therapy 1.6% < patients not on biologic therapy 7.6%
Winthrop et al., 2020 [37]	COVID-19: 2500, of which 77 on immunomodulator therapy (diagnosis: RA, UC, sarcoidosis and others)	Molecular swab (PCR)	Canada	Until 22 May 2020	63 (81.8%) hospitalizations 27 (35.1%) mechanical ventilations 37 (48.1%) ICU 9 (11.7%) deaths	To report COVID-19 cases among patients assuming immunomodulatory therapies	Hospitalization required in 50% of patients on anti-TNF therapy 73.3% of patients on biologic (non anti-TNFs) therapy 90.9% of patients on DMARDs therapy 100% of patients od DMARDs + corticosteroids or only corticosteroids 66.7% of patients on JAK inhibitors treatment 0 deaths among patients on anti-TNF therapy
Rizzello et al., 2020 [12]	IBD: 1158 COVID-19: 26 (2.2%)	Molecular swab (PCR)	Italy	10 March 2020–10 June 2020	521 patients on biologic therapy. Treatment interrupted in 85 patients and delayed in 195. Worsening of symptoms in 200 patients on biologic therapy (189 interrupted it)	To understand the incidence of COVID-19 between IBD patients and to evaluate possible risk factors for the infection	5 patients on biologics, 16 on mesalazine, 5 on corticosteroids and 1 on thiopurines Hospitalization in 7 patients (none was in biologic therapy) 2 deaths (mesalazine therapy) Anti-TNFs could reduce the infection severity The continuous corticosteroids treatment could represent a risk factor for the infection
Burke et al., 2020 [38]	IBD: 5302 COVID-19: 39 (0.7%)	Molecular swab (PCR)	Massachusetts	01 January 2020–25 April 2020	7 hospitalized patients 3 ICU 1 death	To clarify the effect of biologics and immunomodulators on COVID-19 risk	Infection: 0.64% of patients on mesalamine/sulfasalazine therapy, 0.5% of them on immunomodulators, 1% of patients on anti-TNFs, 1% among patients on Vedolizumab therapy. Drug intake does not influence infection risk. Corticosteroids’ or other immunosuppressor’s use have not been associated with a higher infection risk (users 0.37% vs. nonusers 0.36% regarding corticosteroids)
Kennedy et al., 2021 [39]	IBD: 6935 (patients ≥ 5 years under Infliximab or Vedolizumab treatment for at least 6 weeks)	Certain cases: molecular swab (PCR) Highly suspected cases: clinic	UK	22 September 2020–23 December 2020	Anti-SARS-CoV-2 antibodies: 4.3% (295)	To study if IBD patients under Infliximab treatment show reduced serological response to the infection	Anti-SARS-CoV-2: 3.4% of patients under Infliximab (161/4685) < 6% of patients under Vedolizumab (134/2250), *p* < 0.0001 Infliximab is associated to a lower seropositivity level compared to Vedolizumab (OR:0.66, *p* = 0.0027) or other immunomodulators (OR:0.70, *p* = 0.012) Among patients with COVID-19 confirmed diagnosis, seroconversion regarded: Infliximab (48%, 39/81) < Vedolizumab (83%, 30/36, *p* = 0.00044) even though the incidence of symptoms was similar in the two groups Failure of seroconversion has been linked to the concomitant use of immunomodulators: in patients treated with Infliximab only the seroconversion rate was 60% (24/40) while in patients treated with infliximab and immunomodulators it was 37% (15/41, *p* = 0.046)
Bossa et al., 2021 [40]	IBD: 259 (27 children) Controls: 214 non-IBD patients	Serologic test	Foggia (Italy)	February 2020–June 2020	Infection rate (0.77) comparable to general population (0.19), *p* = 0.5 32 patients (12.3%) developed COVID-like symptoms (1 of them under Infliximab therapy)2 hospitalizations	To understand the impact of SARS-CoV-2 infection in IBD patients and the serum prevalence of antibodies in IBD patients under biologics	Seroprevalence (anti SARS-CoV-2 antibodies) comparable between IBD patients (0.77) and general population (0.9) No risk associated with biologic therapy (34.4% Adalimumab, 24% Infliximab, 22% Vedolizumab, 10.4% Ustekinumab, 7.7% Golimumab, 1.1% experimental therapy, 0.4% thalidomide)
Khan et al., 2021 [41]	IBD: 30,911 COVID-19: 649	Molecular swab (PCR)	USA, VAHS database	20 January 2020–10 December 2020	125 hospitalizations 41 deaths	To understand the role of IBD therapies in the risk of infection and their impact in the infection course	Vedolizumab is associated with a greater infection risk than mesalazine (HR:1.70, *p* = 0.006) Corticosteroids are associated with an increased risk of infection and of severe forms (hospitalization or death) No differences in terms of outcome between patients on mesalazine and on anti-TNFs Patients who are not under therapy have a significatively higher risk of severe infection compared to patients under mesalazine
Berte et al., 2020 [42]	IBD: 354 (biologic therapy) COVID-19 IgG: 8	Serologic test	Italy and Germany	April 2020–June 2020	Anti-SARS-CoV-2 IgG have been found in 8 patients (higher incidence of symptoms and contact with positives in these patients)	To determine SARS-CoV-2 infection prevalence in IBD patients under biologics	Seroprevalence (IgG anti SARS-CoV-2) in IBD patients on biologic therapy comparable to that found in general population (Milan 7.5%, Sardinia 0.3% e Germany 0.9%)
Agrawal et al., 2021 [43]	IBD and COVID-19: 3647 patients, of which 457 (12.5%) on Vedolizumab therapy	Laboratory diagnosis	Data from SECURE-IBD database	Until 26 January 2021	664 hospitalizations 166 severe forms of infection (ICU admission, mechanical ventilation and/or death)	To study Vedolizumab effects in IBD patients who undergo COVID-19	Vedolizumab is safe and it is not associated with hospitalizations or more severe infections compared to other drugs (aOR:0.87 e 0.95) Hospitalization risk (but not the risk of severe forms) is increased in patients on Vedolizumab monotherapy compared to patients on anti-TNFs (aOR:1.38, aOR:2.92, *p* = 0.049 e *p* = 0.055)
Brenner et al., 2020 [44]	IBD and COVID-19: 525 (age ≥ 5)	Laboratory diagnosis	Data from SECURE-IBD database	Until May 2020	Severe forms in 37 patients (ICU, mechanical ventilation, death) Only 3 pediatric patients (10%) hospitalized (none of them in ICU)	To study the clinical course of COVID-19 in IBD patients and to find eventual associations with clinical and demographic characteristics and with immunosuppressant treatment	Factors that have been connected to severe forms: advanced age, comorbidities, use of systemic corticosteroids (aOR:6.9), sulfasalazine (aOR:3.1) Anti-TNFs (43.4% of patients) are not associated with severe forms (aOR:0.9)
Allocca et al., 2020 [20]	IBD: 23,879 COVID-19: 97	Molecular swab in 64 patients Highly suspected (clinic + contact or radiology) 33 patients	Italy, United Kingdom, France, Spain, Portugal, Malta, Kastoria, Attica, Greece, Russia and Israel	21 February 2020–30 June 2020	Symptoms in 90% of positives. Pneumonia in 22%. Hospitalization in 24%. 1 death	To study the incidence of COVID-19 and the eventual effects of immunosuppression on the risk of infection	Corticosteroids treatment is associated with an increased risk of hospitalization (OR 7.69, *p* = 0.015), while the treatment with monoclonal antibodies is associated with a reduced risk of pneumonia and hospitalization (OR 0.15, *p* = 0.003 e OR 0.31, *p* = 0.031).
Norsa et al., 2020 [21]	IBD: 522, of which 59 < 18 aa Controls with COVID-19: 479	Molecular swab	Hospital “Papa Giovanni XXIII”, Bergamo (Italy)	19 February 2020–23 March 2020		To report the experience of this IBD Italian center during the pandemic	0 COVID-19 cases among IBD patients (despite the therapy with immunomodulators in 22% of them and biologics in 16%)
Hormati et al., 2020 [45]	IBD (or AIH): 200 (treated with Azathioprine, anti-TNFs and prednisone) COVID-19: 11 (8 with IBD)	Molecular swab	Iran	Since the beginning of pandemic (publication date): 28 May 2020)	Only mild symptoms that disappears more rapidly compared to general population, as like the RX alterations. 0 deaths	To study the effects of immunosuppressive drugs in SARS-CoV-2 infection	Percentage of positives lower than the general population not receiving immunosuppressors
Lukin et al., 2020 [24]	IBD and COVID-19: 80 (considered positive with both a molecular test or highly suspect clinic) COVID-19 non IBD: 160	Molecular swab or highly suspect clinic	New York	01 February 2020–30 April 2020		To notice eventual differences between COVID-19 patients with and without IBD in terms of clinical outcomes and to study risk factors for COVID-19 in IBD patients	Use of corticosteroids is higher among patients with COVID-19 than in patients with IBD but not infected No differences regarding biologics and immunomodulators The proportion of patients receiving Vedolizumab or not receiving biological therapy was numerically higher in patients requiring hospitalization (no biologics: 29%, Vedolizumab: 30%, Ustekinumab 8%, anti TNFs (6%) *p* = 0.197) compared to others
Khan et al., 2020 [46]	IBD: 37,857 COVID-19: 36	Laboratory diagnosis	USA, VAHS database	01 January 2020–15 May 2020	2391 patients on thiopurine therapy: 2 COVID-19 4920 patients on anti-TNF therapy: 3 COVID-19	To study the impact of anti-TNF and thiopurines on SARS-CoV-2 infection	Thiopurines are not connected to an increased risk of infection (OR:0.962, *p* = 0.9577) Anti-TNFs are not associated with an increased risk of infection (OR:0.581, *p* = 0.3774)
Singh et al., 2020 [27]	IBD: 196,403 COVID-19: 232 (1901 tests) Controls: 19,776 COVID-19	Laboratory diagnosis or diagnostic code for COVID-19 after hospitalization	USA	26 January 2020–26 May 2020	Risk of severe infection (hospitalization and/or death within 30 days after diagnosis) similar between IBD patients and controls	To study clinical presentation and outcomes of COVID-19 among IBD patients and compare them to a large control group	Higher risk of severe forms among patients under corticosteroids treatment for at least 3 months (30.98%) compared to other patients (19.25%) RR:1.6, *p* = 0.4 (univariate analysis)
Allocca, Guidelli et al., 2020 [47]	COVID-19: 41 patients with immune mediated inflammatory diseases (IMID). Among them 12 UC and 9 CD	Molecular swab or highly suspect clinic or chest CT	Italy	N.R.	All patients developed symptoms due to infection: 16 pneumonia (40%), 14 hospitalizations (34%), 0 ICU admissions and 1 death	To report the experience of the Humanitas center (Milan) among patients with IMID	Corticosteroids therapy increases risk of oxygen therapy needing (*p* = 0.007) Biologics are not associated with hospitalization risk

IBD: inflammatory bowel disease. TNF: tumor necrosis factor. DMARDs: disease modifying antirheumatic drugs. CS: corticosteroids. RA: rheumatoid arthritis. UC: ulcerative colitis. JAK: Janus kinase. ICU: intensive care unit. AIH: autoimmune hepatitis. IMID: immune mediated inflammatory diseases. CD: Crohn’s disease. UC: ulcerative colitis. PCR: polymerase chain reaction. TC: computed tomography. ICU: intensive care unit.

## Data Availability

Not applicable.
